# Wavefront Derived Refraction and Full Eye Biometry in Pseudophakic Eyes

**DOI:** 10.1371/journal.pone.0152293

**Published:** 2016-03-24

**Authors:** Xinjie Mao, James T. Banta, Bilian Ke, Hong Jiang, Jichang He, Che Liu, Jianhua Wang

**Affiliations:** 1 School of Ophthalmology and Optometry, Wenzhou Medical University, Wenzhou, China; 2 Bascom Palmer Eye Institute, University of Miami Miller School of Medicine, Miami, FL, United States of America; 3 Department of Ophthalmology, Shanghai First People's Hospital, Shanghai Jiao Tong University, Shanghai, China; 4 New England College of Optometry, Boston, Massachusetts, United States of America; Charité University Medicine Berlin, GERMANY

## Abstract

**Purpose:**

To assess wavefront derived refraction and full eye biometry including ciliary muscle dimension and full eye axial geometry in pseudophakic eyes using spectral domain OCT equipped with a Shack-Hartmann wavefront sensor.

**Methods:**

Twenty-eight adult subjects (32 pseudophakic eyes) having recently undergone cataract surgery were enrolled in this study. A custom system combining two optical coherence tomography systems with a Shack-Hartmann wavefront sensor was constructed to image and monitor changes in whole eye biometry, the ciliary muscle and ocular aberration in the pseudophakic eye. A Badal optical channel and a visual target aligning with the wavefront sensor were incorporated into the system for measuring the wavefront-derived refraction. The imaging acquisition was performed twice. The coefficients of repeatability (CoR) and intraclass correlation coefficient (ICC) were calculated.

**Results:**

Images were acquired and processed successfully in all patients. No significant difference was detected between repeated measurements of ciliary muscle dimension, full-eye biometry or defocus aberration. The CoR of full-eye biometry ranged from 0.36% to 3.04% and the ICC ranged from 0.981 to 0.999. The CoR for ciliary muscle dimensions ranged from 12.2% to 41.6% and the ICC ranged from 0.767 to 0.919. The defocus aberrations of the two measurements were 0.443 ± 0.534 D and 0.447 ± 0.586 D and the ICC was 0.951.

**Conclusions:**

The combined system is capable of measuring full eye biometry and refraction with good repeatability. The system is suitable for future investigation of pseudoaccommodation in the pseudophakic eye.

## Introduction

Monofocal intraocular lens (IOL) implantation during cataract surgery often provides good uncorrected distance vision, but middle and near visions are rarely satisfactory without correction [1]. However, in some instances, pseudophakic patients with monofocal IOLs maintain good uncorrected near visual acuity along with good uncorrected distance visual acuity also. This phenomenon is often referred to as pseudoaccommodation and is attributed to several factors such as corneal aberration or multifocal function of the cornea [2], depth of field change [3], change of IOL position and cortical mechanisms [4]. IOL position change is hypothesized to result from IOL movement in response to ciliary muscle contraction [5]. Despite extensive research, the mechanisms of pseudoaccommodation remain controversial. To fully understand pseudoaccommodation mechanisms, a combined system that can image the geometrical structure of the eye, the ciliary muscle and optical performance is very valuable. This type of multifunction system has been previously unavailable, possibly due to the technical difficulty of combining all necessary imaging modalities into one system.

Several imaging techniques have been previously used to investigate the ocular biometry and IOL position in the pseudophakic eye including magnetic resonance imaging (MRI) [6], Purkinje imaging [7], ultrasound biomicroscopy [8], laser interferometry [9,10] and optical coherence tomography (OCT) [11]. Imaging ocular parameters for the pseudophakic eyes, however, is not straightforward. For example, the A-scan laser interferometry (IOL Master) has been widely used for normal people, but it could not produce good measurement for pseudophakic patients in most cases. Recent studies have demonstrated that full eye imaging can be achieved by B-scan OCT with prolonged scan depth, while the high resolution remains [12–14]. Inspired by a previous study using a switchable reference for prolonging OCT scan depth [15], we have recently reported a custom-built OCT for full-eye imaging utilizing a reference arm of four mirrors that can image the entire eye and obtain biometry from cornea to retina [16–18]. A similar approach was also used in other studies [11,19]. We have validated the measurement of the fully eye biometry in phakic eyes [16–18]. Meanwhile, a spectral domain OCT (SD-OCT) with a 1310nm light source was utilized to image the ciliary muscle [17]. In the present study, we further improved the system by integrating a Shack-Hartmann wavefront sensor into the system. The goal was to validate the measurements of whole eye biometry, ciliary muscle dimension and wavefront-derived refraction for a non-accommodative state in the pseudophakic eye.

## Methods

This study followed the Declaration of Helsinki rules and was reviewed and approved by the Institutional Review Board for Human Research at the University of Miami (#20070492). After explaining the nature of the study, each patient signed a written consent form before being enrolled. This consent procedure was approved by the IRBs. There were 28 adult subjects (10 males and 18 females) enrolled in this study and 32 eyes were imaged. All subjects were recruited from the Bascom Palmer Eye Institute after undergoing cataract surgery. A 1-Piece Acrylic IOL (Tecnis^®^, Abbott Medical Optics Inc., USA) was implanted in all 32 eyes.

In the previously reported version of the imaging system [17,20,21], we combined one SD-OCT utilizing a complementary metal oxide semiconductor (CMOS) camera (CMOS-OCT) with long scan depth to obtain full images from the cornea to the retina and second SD-OCT system with a light source of 1,310 nm for primarily imaging the ciliary muscle (ciliary muscle-OCT or CM-OCT). A model eye (OEMI-7, Ocular^®^ Instruments Inc., Bellevue, WA, USA) and an IOL (AcrySof^®^ Natural single-piece acrylic foldable IOL; Alcon, USA) were utilized to validate OCT full eye biometry. The model eye was made from polymethyl methacrylate (PMMA) and the model crystalline lens was replaced with the IOL. Saline solution was used to fill the model’s anterior chamber and vitreous chamber. The central corneal thickness (CCT), anterior chamber depth (ACD), IOL thickness (IOLT), vitreous length (VL) and axial length (AL) of the model eye were measured and calculated using a digital caliper (Tresna; Guilin Guanglu Measuring Instrument Co Ltd, Guangxi Province, China), a digital thickness gauge (IK-C1012EBS; Mitutoyo Corp, Takatsuku, Kawasaki, Kanagawa, Japan) and an electronic thickness gauge (Model ET-3; Rehder Development Corp, Castro Valley, CA, USA) as previously reported [16]. The refractive indices used in calculating the axial biometry of the IOL model eye using ultra-long scan depth OCT (wavelength = 840nm) were 1.483 for polymethyl methacrylate, 1.336 for saline solution, and 1.55 for the acrylic IOL (adapted for use with the visible wavelength). All measured parameters obtained by CMOS- OCT highly correlated with the geometry of model eye and IOL.

In this study, we further integrated a Shack-Harmann wavefront sensor into the system. Similar to the system reported in a previous study [22], the Shack-Harmann wavefront sensor has a superluminescent diode (SLD) light (TO-56, Langhorne, PA, USA) of 750 nm wavelength and 25 nm bandwidth. Light with an output power of 0.2mW reaches the fovea after passing through a collimator, a pellicle beam splitter (Thorlabs Inc, Newton, NJ, USA) a dichroic beam mirror (Thorlabs Inc, Newton, NJ, USA) and the optical system of the eye. A sensor receives the reflected light from the retina, and a 10 × 10 microlens array (Edmund Optics, Barrington, NJ, USA) in the sensor monitors the local refractive deviation caused by the wavefront aberrations. Each microlens has dimension of 0.5 mm × 0.5 mm with a focal length of 32.8 mm. A charge-coupled device (CCD) camera (Uniq Vision, Santa Clara, CA, USA) behind the microlens array captures the distorted wave front pattern of the eye. A Badal system with a visual target provides automatic correction of the subject’s refractive error and further provides an accommodative stimulus of up to eight diopters using a removable, adjustable lens (Thorlabs, Newton, NJ, USA). Movement of the Badal system was measured and accommodation stimulus was calculated using the conversion 1 cm of movement equals 1D of accommodation. The visual target was a white “Snellen E” with black background printed on a glazed transparent paper, backlit by a miniature LED bulb with dim illumination. A combined optical system of a model eye (OEMI-7, Ocular^®^ Instruments Inc., Bellevue, WA, USA) and a series of trial lenses were used to calibrate the wavefront sensor. The Zernike defocus term measured by the optical system was highly correlated to the trial lens power with a correlation coefficient of 0.985. The camera was controlled by a custom software package, and wavefront images were acquired at 60 frames per second.

**Fig 1:**
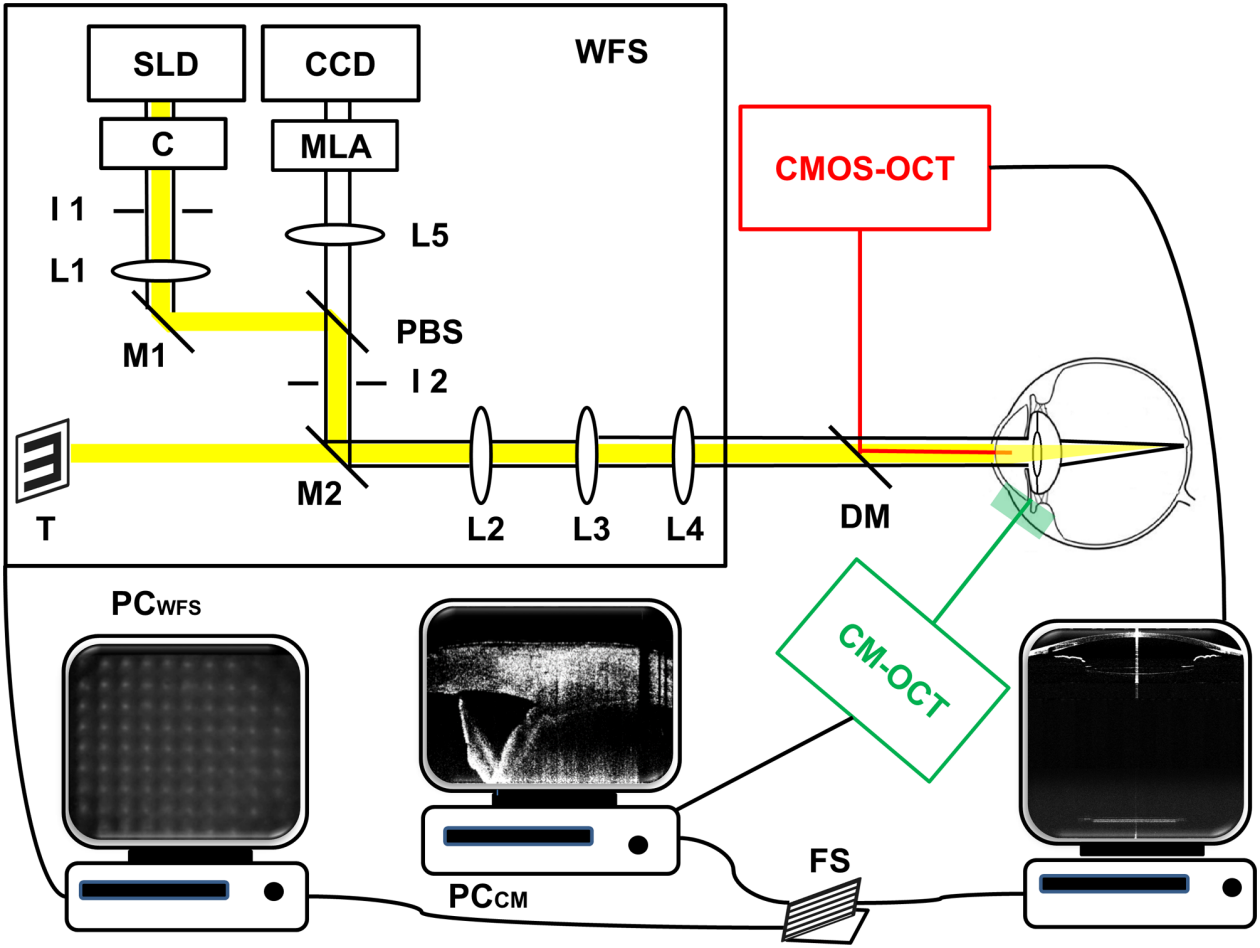
Schematic of the synchronic system. SLD: superluminescent diode with a central wavelength of 750 nm; C: collimator; I: Iris; M: mirror; PBS: pellicle beam splitter; L: lens; DM: dichroic mirror; MLA: microlens arrays; CCD: CCD camera; T: visual fixation target; PC: personal computer; FS: foot switch; WFS: wavefront sensor.

The probes of the CMOS-OCT, CM-OCT and Shack-Hartmann wavefront sensor were mounted on a slit-lamp with a trinity sample arm. Fig 1 shows the schematic of this synchronic system. The apices of the horizontal and vertical frames by CMOS-OCT cross-section scanning were used to align the eye location [17]. The apex of the CM-OCT and Shack-Hartmann wavefront sensor was pre-aligned with the CMOS-OCT system before the experiment.

The subjects were asked to sit in front of the probe with their chin and forehead against the rests and fix on the target “E” with the subject eye. The non-study eye was occluded using a cover mounted on the slit-lamp headrest. The adjustable lens of the Badal system was placed at the far end of the scale, blurring the target “E”. The adjustable lens was slowly moved towards the patient and stopped when the subject first reported the target “E” was clear. This position was utilized as the subject’s far point (0D accommodation stimulus). The axial biometry of the eye and the temporal ciliary muscle of the left eye or the nasal ciliary muscle of the right eye were imaged by the combined imaging system. All measurements were performed with the pupil pharmacologically dilated, and the test was repeated twice within 15 minutes.

The imaging acquisition procedure of the CMOS-OCT and CM-OCT was the same as previously reported [17,18]. Each imaging acquisition obtained a set of corresponding images as follows: 640 pixels × 480 pixels Shack-Hartmann spot pattern image in 0.02 seconds; 2,048 pixels × 4,096 pixels × 4 frames of the whole eye CMOS-OCT images in 0.47 s at 17,500 A-lines/second; 1,000 pixels × 1,223 pixels ciliary muscle image in 0.12 s at 7,000 A-lines/second.

The image analysis methods used with the CMOS-OCT and CM-OCT were described in previous studies [17,18]. Image processing data of anterior chamber depth (ACD), central IOL thickness (IOLT), vitreous length (VL), and axial length (AL) were obtained from the CMOS-OCT. Values of ciliary muscle thickness at 1 mm (CMT1), 2 mm (CMT2), 3 mm (CMT3), maximum thickness of the ciliary muscle posterior to the scleral spur (CMTM), and anterior length of the ciliary muscle (CAL) were obtained from the CM-OCT images. Two ciliary muscle images were superimposed to enhance the performance and resultant images.

Wavefront spot patterns of the central 9 × 9 points, representative of a central 4-mm diameter pupil, were analyzed using a custom-developed code [23]. Zernike coefficients of defocus aberration (Z(2,0)), low order aberration (LOA) and high order aberration (HOA) were calculated. Equivalent defocus was defined as the amount of defocus related to the normalized Zernike coefficients by the following equations [24]:

$$\text{M}=\frac{-Z_{2}^{0}4\sqrt{3}}{r^{2}},$$

where the r represents the radius of the pupil. The result of each session was an average of five wavefront images displayed in one image as a color-coded map of higher order aberrations based on each series of Zernike coefficients.

All data were presented as the mean ± standard deviation and a paired t test was used in statistical analysis. P <0.05 was considered significant. The coefficient of repeatability (CoR) was defined as 2 standard deviations of the difference between two measurements. The CoR% was calculated as CoR divided by the average of the two measurements performed on each eye.[25]

## Results

**Fig 2:**
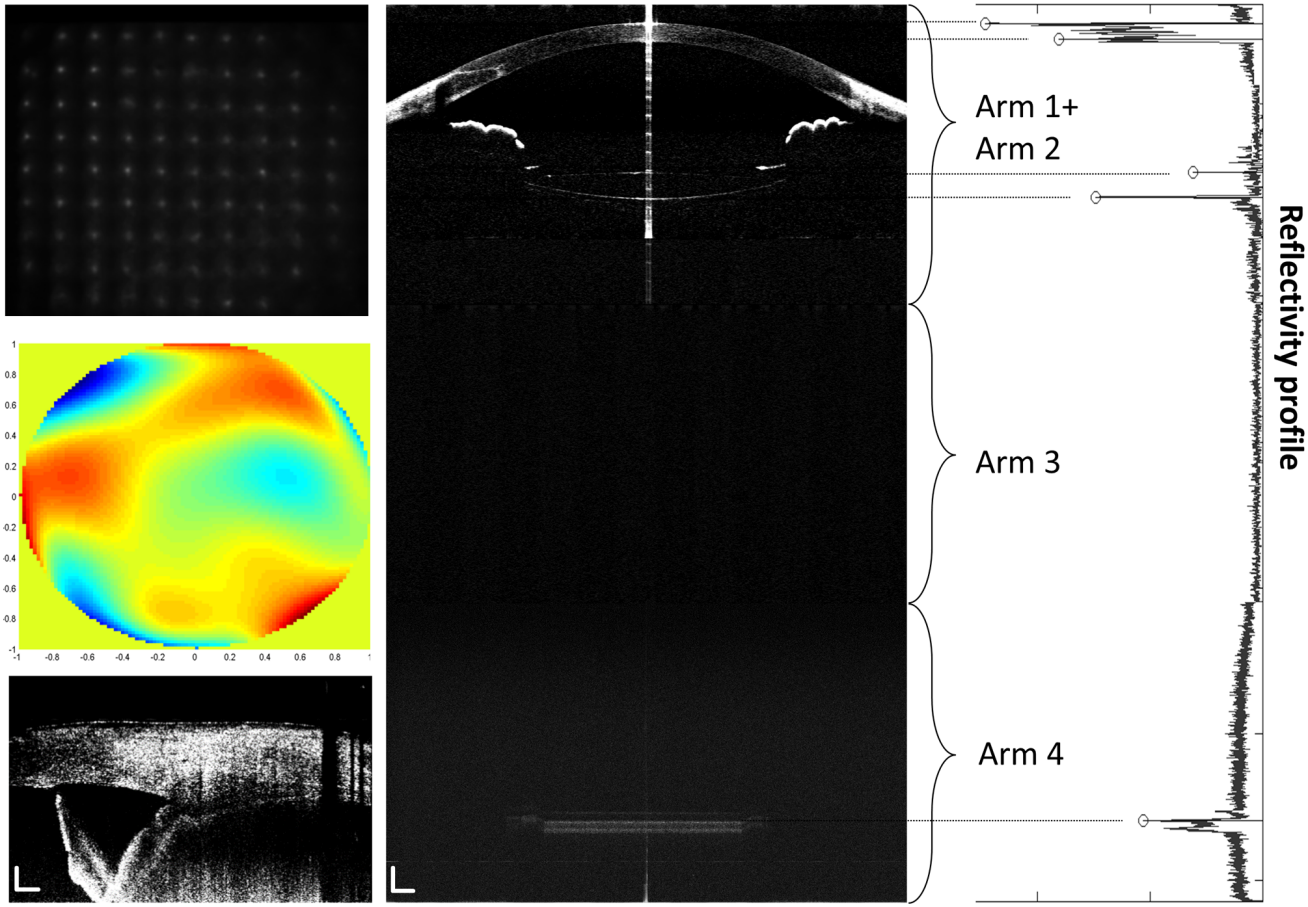
Typical images of a pseudophakic eye obtained with the combination system. Shack-Hartmann wavefront sensor spot patterns with a 0D accommodation stimulus (upper left). Higher order aberration image with a 0D accommodation stimulus (middle left). CM-OCT ciliary muscle image with a 0D accommodation stimulus (lower left). Bars = 500 μm (C) CMOS-OCT whole eye image with a 0D accommodation stimulus (right). Bars = 1000 μm (D).

**Fig 3:**
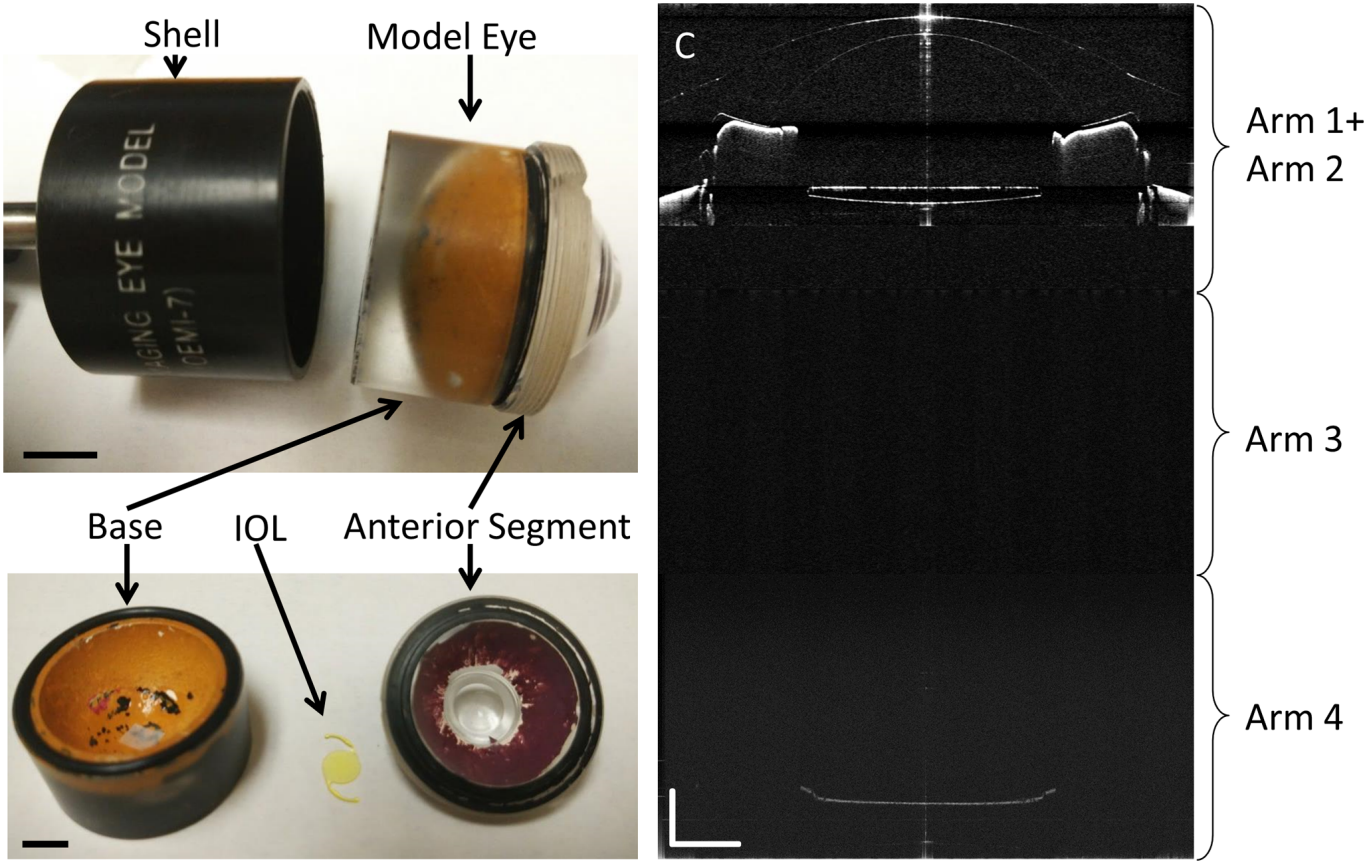
A model eye with an IOL used for calibration. A model eye (upper left) was used to calibrate the measurement. Bar = 1000μm. The crystalline lens in the model eye was replaced with an IOL (lower left). Bar = 500μm. The full eye image (right) obtained with the long scan depth OCT clearly shows the IOL and other structures of the model eye, yielding the OCT axial biometry. Bar = 2000μm.

**Fig 4:**
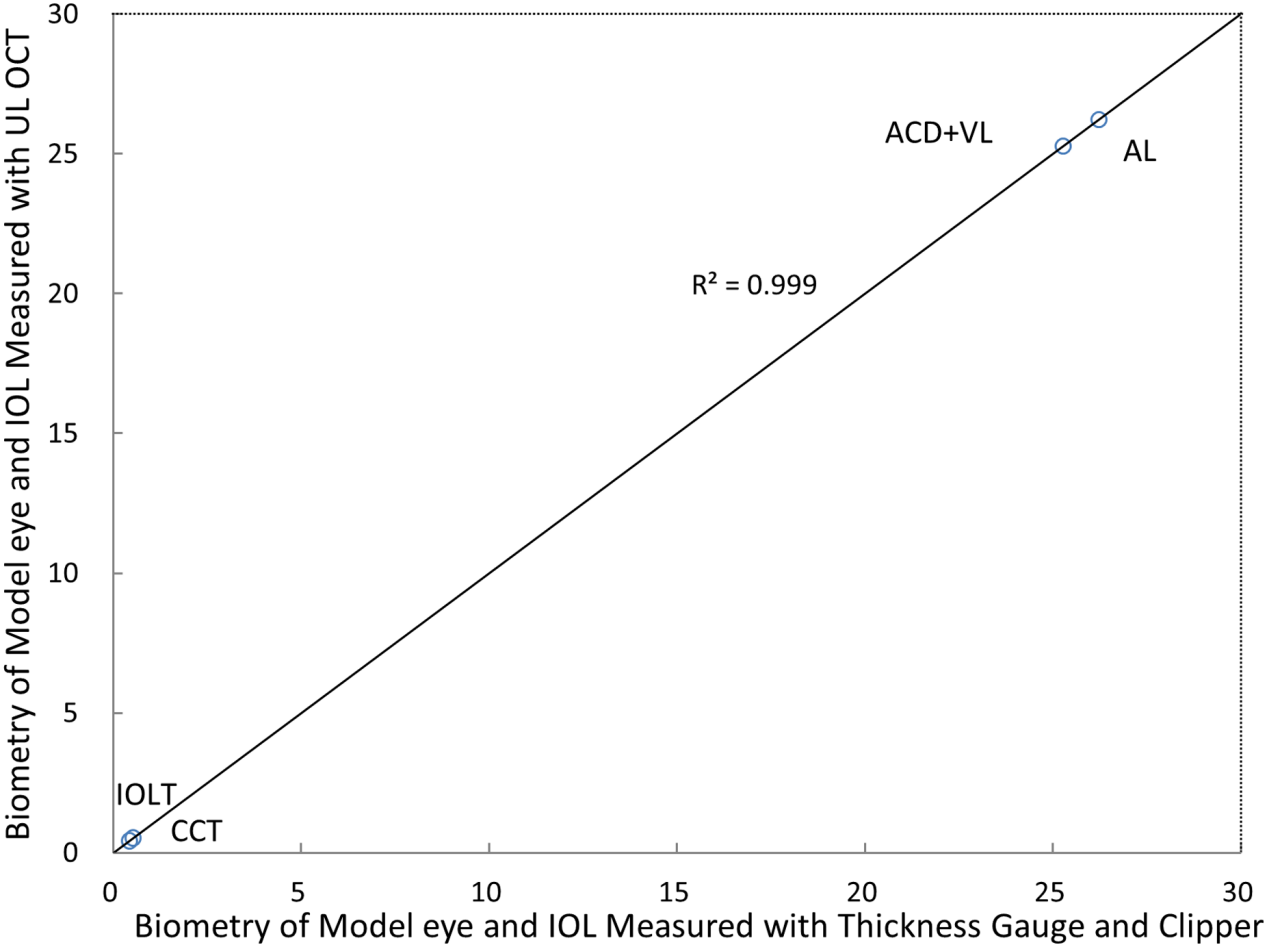
Graph shows the correlation between the axial biometry of the whole eye measured by CMOS-OCT and the actual geometry of the model eye combining an IOL. The solid line represented the equality. The correlation was strong (R^2^ = 0.999; P < .05). **CCT** = central corneal thickness; **IOLT** = IOL thickness; **ACD** = anterior chamber depth; **VL** = vitreous length; **AL** = axial length.

**Fig 5:**
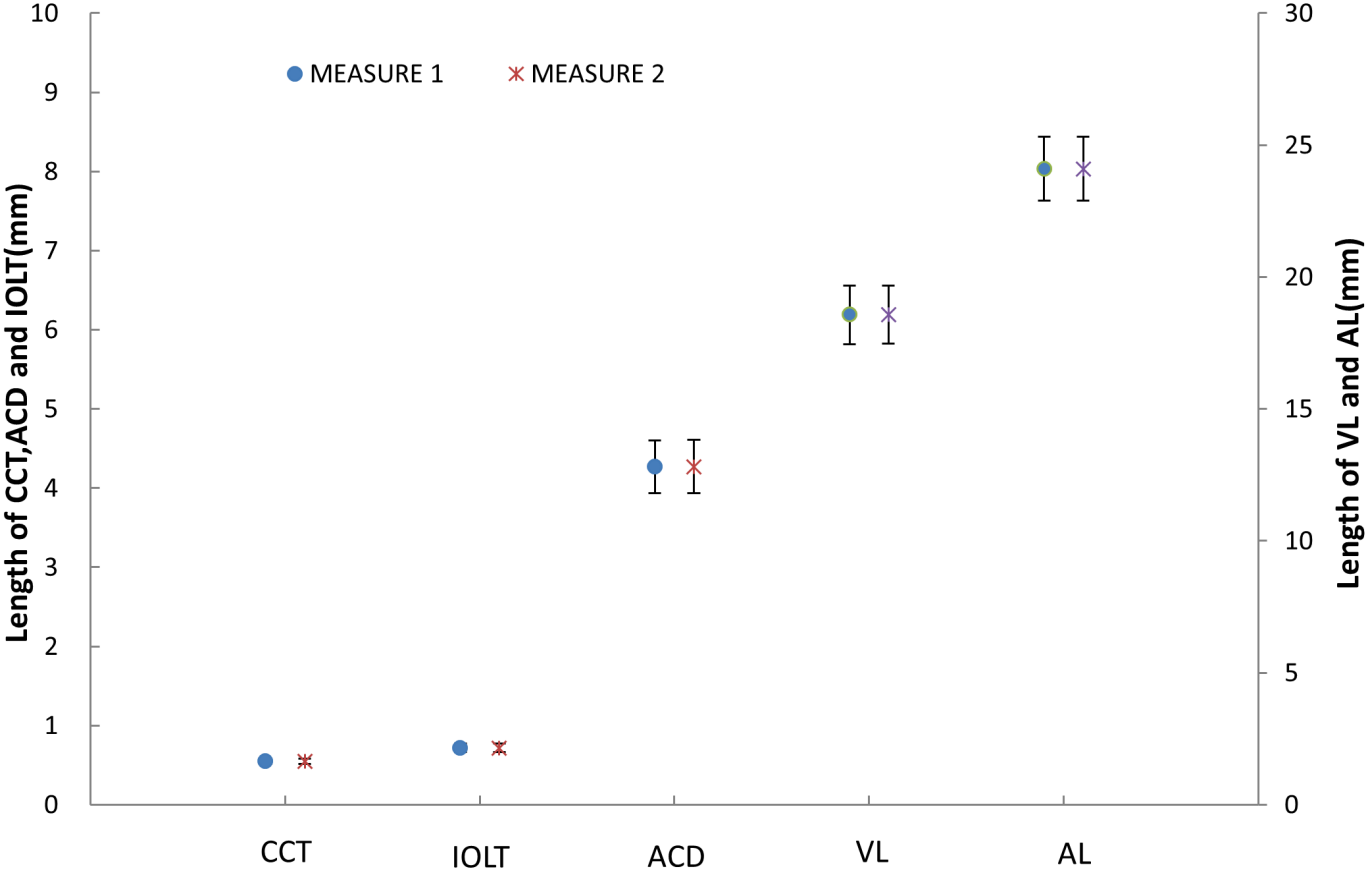
Whole eye biometry within two measurements for IOL patients (n = 32). **CCT** = central corneal thickness; **ACD** = anterior chamber depth; **IOLT** = IOL thickness; **VL** = vitreous length; **AL** = axial length. Bars = standard deviation.

**Fig 6:**
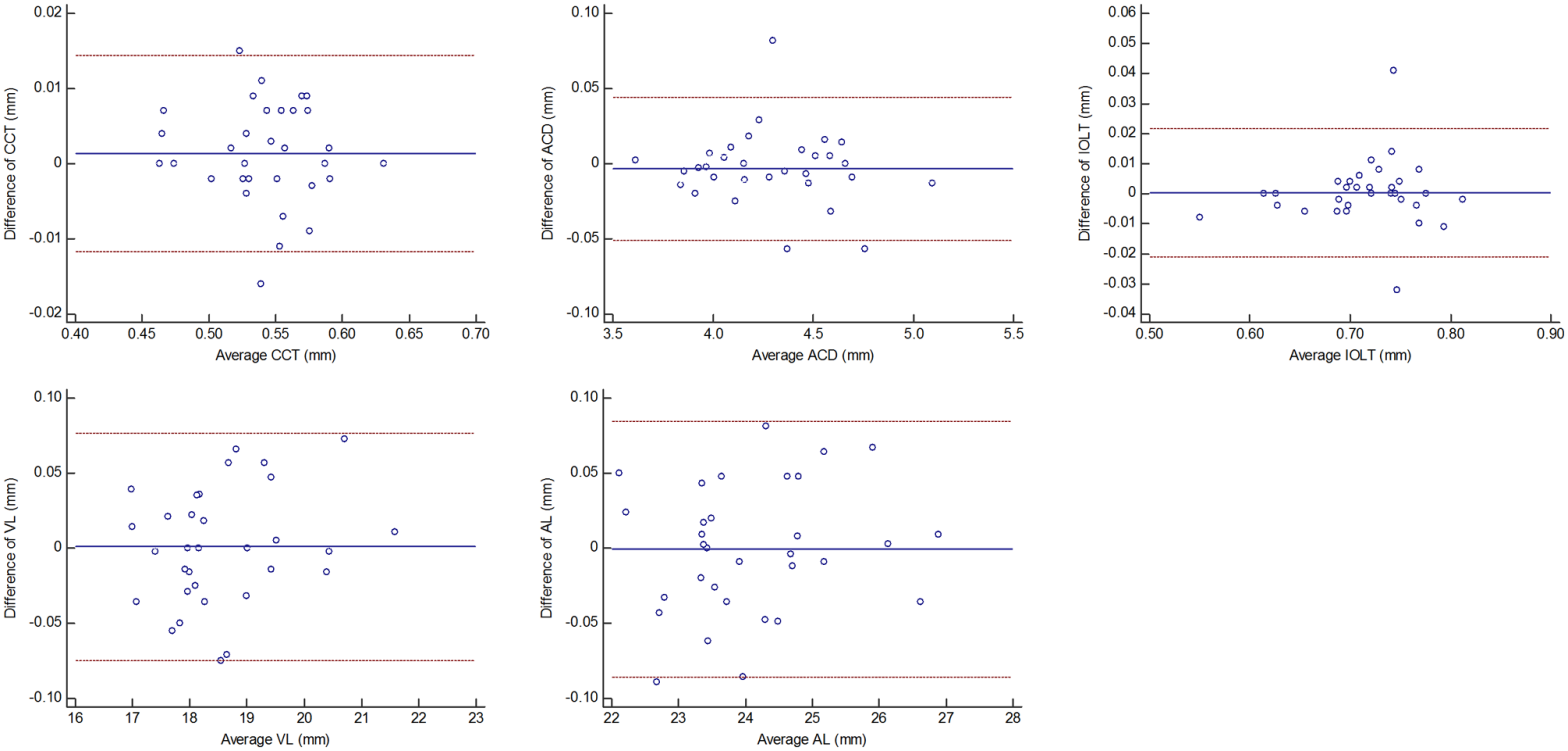
Bland-Altman plots of the difference between the two repeated measurements of full-eye axial biometry in 32 pseudophakic eyes. The full-eye axial biometry included (upper left) the central corneal thickness (CCT), (upper middle) the anterior chamber depth (ACD), (upper right) the IOL thickness (IOLT), (lower left) the vitreous length (VL), and (lower middle) the axial length (AL). Values on the horizontal axis correspond to the mean of the 2 measurements. Values on the vertical axis correspond to the difference of 2 measurements. Solid lines and dashed lines indicate mean differences and 95%limits of agreement.

Fig 2 shows the processed wavefront, CM-OCT and CMOS-OCT images of a 76 year old subject with a 0D accommodative stimulus. Images were successfully acquired in all study eyes and all image parameters were successfully processed. Fig 3 clearly shows the full eye image with the IOL and other structures of the model eye obtained with the long scan depth OCT. The difference between repeated measurements for full biometry of a model eye using CMOS-OCT was plotted (Fig 4). Whole eye biometry within two measurements for IOL patients (n = 32) was shown in Fig 5. The difference between repeated measurements for full biometry of IOL eyes was plotted using a Bland-Altman Plot (Fig 6). The CoRs for CCT, ACD, IOLT, VL and AL were 0.013, 0.049, 0.022, 0.077, and 0.087mm respectively. The percentage of CoR ranged from 0.36% to 3.04% and the ICC ranged from 0.981 to 0.999 (Table 1).

**Table 1:** Axial Biometry of the Full Eye Using Ultra-Long Scan Depth Optical Coherence Tomography (n = 32).

	CCT	ACD	IOLT	VL	AL
**Measure 1 (mm)**	**0.543 ± 0.039**	**4.274 ± 0.330**	**0.715 ± 0.056**	**18.564 ± 1.101**	**24.097 ± 1.193**
**Measure 2 (mm)**	**0.542 ± 0.039**	**4.278 ± 0.333**	**0.715 ± 0.055**	**18.563 ± 1.093**	**24.102 ± 1.186**
**Difference (mm)**	**-0.001 ± 0.007**	**0.003 ± 0.024**	**0.000 ± 0.011**	**-0.001 ± 0.039**	**0.001 ± 0.043**
**CoR(mm)**	**0.013**	**0.049**	**0.022**	**0.077**	**0.087**
**CoR%**	**2.425**	**1.140**	**3.044**	**0.416**	**0.361**
**ICC**	**0.986**	**0.997**	**0.981**	**0.999**	**0.999**

CCT = central corneal thickness; ACD = anterior chamber depth; IOLT = IOL thickness; VL = vitreous length; AL = axial length; CoR = coefficient of repeatability; ICC = intraclass correlation coefficients.

There were no significant differences between the repeated measurements for ciliary muscle using CM-OCT (P > 0.05, Table 2). The CoR of CMT1, CMT2, CMT3, CMTM and CAL were 0.070, 0.074, 0.052, 0.081, and 0.378mm respectively. The percentage of CoR ranged from 12.2% to 41.6% and the ICC ranged from 0.767 to 0.919 (Table 2).

**Table 2:** Biometry of Ciliary Muscle using CM-OCT (n = 32).

	CMT1	CMT2	CMT3	CMTM	CAL
Measure 1(mm)	0.569 ± 0.088	0.328 ± 0.130	0.171 ± 0.072	0.629 ± 0.079	0.905 ± 0.309
Measure 2 (mm)	0.574 ± 0.092	0.315 ± 0.117	0.172 ± 0.063	0.634 ± 0.068	0.910 ± 0.266
Difference (mm)	0.005 ± 0.035	-0.014 ± 0.037	0.002 ± 0.026	0.004 ± 0.040	0.006 ± 0.189
CoR (mm)	0.070	0.074	0.052	0.081	0.378
CoR %	12.220	22.841	30.466	12.788	41.603
ICC	0.767	0.919	0.792	0.804	0.812

CMT1,2,3 = Ciliary Muscle Thickness at 1 mm, 2 mm and 3 mm posterior to the scleral spur; CMTM = Maximum Thickness of Ciliary Muscle; CAL: Anterior Length of Ciliary muscle.

There were no significant differences between the repeated measurements for wavefront aberration using the wavefront sensor (P > 0.05). Defocus aberration at a far target was -0.44 ± 0.54D. CoR of the defocus aberration of two measurements was 0.40D and the ICC was 0.951 (Table 3).

**Table 3:** Defocus aberration, low order aberration and high order aberration using wavefront sensor (n = 32).

	DA(D)	LOA (μm)	HOA (μm)
Measure 1 (mm)	0.443 ± 0.534	0.749 ± 0.381	0.387 ± 0.096
Measure 2 (mm)	0.447 ± 0.586	0.744 ± 0.410	0.394 ± 0.100
Difference (mm)	0.004 ± 0.198	-0.005 ± 0.272	0.007 ± 0.073
CoR (mm)	0.396	0.545	0.146
COR%	22.258	18.249	9.370
ICC	0.951	0.766	0.721

DA = Defocus aberration; LOA = Low order aberration; HOA: High order aberration

## Discussion

In this work, there were 28 adult subjects (10 males and 18 females) enrolled in this study and 32 eyes were imaged. All subjects were recruited from the Bascom Palmer Eye Institute after undergoing cataract surgery. The mean age was 69.4 ± 10.2 years (range, 51 to 90 years) and the average duration after surgery was 2.1 months. We developed a combined system that consists of two spectral domain OCT devices and a Shack-Hartmann wavefront sensor for imaging full-eye biometry, ciliary muscle and wavefront aberration in pseudophakic eyes. There are several traditional methods for axial biometry of the whole eye in vivo such as A-scan ultrasound [26,27], radiograph [28], magnetic resonance imaging [29], computed tomography [30], laser Doppler interferometry and optical low-coherence reflectometry [31,32]. A-scan ultrasound is the gold standard for measuring axial length, even though it has limitations including low precision, the need for topical anesthesia and direct contact with the eye [26,27]. Laser interferometry systems such as IOLMaster and Lenstar (LS 900, Haag-Streit AG, Koeniz, Switzerland) use a non-contact imaging method that provides axial measurement in a single dimension using the group index for converting optical length to geometrical length [9,10]. This measurement may be influenced by changes in lens parameters resulting from accommodation [33]. OCT is a non-contact optical and cross-sectional imaging tool that is primarily used for imaging the posterior segment of the eye, including the retina, macula and optic nerve head. Recently, OCT imaging of the anterior segment of the eye, including the cornea, anterior chamber and lens, has become more commonplace. Commercially available OCT units typically cannot image the entire eye because of limited scan depth range and signal drop-off. With advances in OCT scan depth, several recent studies have successfully imaged and measured the entire eye [11,19,34,35]. In our previous study, automatic axial biometry using ultra-long scan depth OCT successfully measured each segment of phakic eyes with good repeatability (CoR ranging from 0.014 to 0.079 mm) [16]. The present study demonstrates the repeatability and accuracy of ultra-long OCT whole eye biometry in pseudophakic eyes. The CoR of AL is 0.086 mm in the present study, slightly higher than that obtained in phakic eyes [16]. Our current pseudophakic subject population was measurably older (mean age = 69) than the population measured in the previous study (mean age = 36), although it is unclear whether this has any bearing on the results.

A Hartmann-Shack sensor, a highly accurate system for measuring ocular wavefront aberration [36], was incorporated into the OCT system utilized in this study to evaluate the optical performance of human eye. The wavefront sensor also provided a methodology for objective accommodation measurement to evaluate any accommodation response [22,23]. In the present study, a Badal system combined with the wavefront sensor was aligned with the optical axis and utilized to determine the far point of the eye (0D accommodation) prior to performing aberration measurements. The wavefront-derived refraction was calculated from the coefficient of the second order of aberration (defocus: Z (2,0)). The subjects in the current study tended to over focus for a distance target inside the Badal system, in agreement with a previous study [37]. The CoR for defocus aberration (0.351D) at a distance target indicated good repeatability. Contraction of the ciliary muscle is considered the launching point of accommodation. The 1310nm CM-OCT system combined with CMOS-OCT is able to clearly image the entire apparatus thought responsible for pseudoaccommodation including the cornea, anterior chamber, IOL, ciliary muscle and retina. The thickness of CMT1, CMT2, CMT3, CMTM and CAL was within the range of previous studies [17,38,39].

OCT has been previously utilized to measure ACD, Pupil Diameter and changes in IOL tilt when accommodation demand is increased in pseudophakic eyes [40,41]. Wavefront sensors have also been used to demonstrate the optical changes in pseudophakic eyes. Anterior segment biometry and wavefront aberration were previously studied combining a custom-built long scan depth OCT with a Shack-Hartmann wavefront sensor [42,43]. However, axial length and ciliary muscle measurements were not demonstrated. To the best of our knowledge, the current study is the first to demonstrate the measurement of whole eye biometry, ciliary muscle dimensions and wavefront-derived refraction for a non-accommodative state in pseudophakic eyes. With further validation on measuring dynamic changes using the combined system, the system could be used widely in accommodation studies, such as measuring accommodation response in aphakic eyes, measuring pseudoaccommodation in pseudophakic eyes, and exploring the mechanisms of myopia development.

This study demonstrates the suitability of the combined system for imaging the pseudophakic eyes. There were several limitations in this study. First, all measurements were performed by the same researcher, so inter-operator reproducibility was not tested. Second, the retinal image obtained with the custom OCT system was not as detailed as that obtained by typical posterior segment OCT devices. The retinal thickness was not measured.

In conclusion, the combination of two SD-OCT devices equipped with a Shack-Hartmann wavefront sensor allows successful and repeatable measurements of full-eye biometry, ciliary muscle configuration and defocus aberration in the pseudophakic eye. It appears that the system is suitable for future clinical studies on accommodation.
